# Comparative Study of Antimicrobial, Anti-Inflammatory, and Antioxidant Activities of Different Parts from *Pterocarpus Santalinoides* l'Her. Ex. DC (Fabaceae)

**DOI:** 10.1155/2021/8938534

**Published:** 2021-12-17

**Authors:** Aimé Cézaire Ayéna, Kokou Anani, Kossi Dosseh, Amegnona Agbonon, Messanvi Gbeassor

**Affiliations:** ^1^Faculty of Sciences and Techniques of the University of Abomey-Calavi, 01 BP 526, Godomey, Benin; ^2^Center for Research and Training on Medicinal Plants (CERFOPLAM), University of Lomé-Togo, BP 1515, Lomé, Togo

## Abstract

**Aims:**

*Pterocarpus santalinoides* is used in Beninese folk medicine for treatment of gastroenteritis. This study aims to compare the antimicrobial, antioxidant, and anti-inflammatory activity of the hydroalcoholic extracts of the leaves, trunk bark, and root.

**Materials and Methods:**

The antimicrobial activity was evaluated by the broth microdilution method on 06 bacterial strains including 03 wild-type strains (*Escherichia. coli* 0157H, *Salmonella* sp., and *Staphylococcus aureus* sp.) and 03 reference strains (*E. coli* ATCC 25922, *S. aureus* ATCC 29213, and *Pseudomonas aeruginosa* ATCC 27853), whereas the anti-inflammatory activity was performed by the carrageenan-induced paw edema method on rats. The DPPH-free radical scavenging was used to determine the antioxidant activity.

**Results:**

The MICs of the leaf extracts varied from 6.25 to 25 mg/mL for all strains. The MICs of the stem bark extracts were 6.5 to 25 *μ*g/mL for five strains (*E. coli* 0157H, *S. aureus* ATCC 25922, *Salmonella* sp., *E. coli* ATCC 25922, and *P. aeruginosa* ATCC 27853) and 3.125 mg/mL for *S. aureus*. Concerning the root extracts, the MICs varied from 12.5 to 50 mg/mL. The best anti-inflammatory power was obtained with the stem bark extract with the percentages of inhibition of 36.09%, 38.98%, and 39.50%. The DPPH test showed that the hydroethanolic extract of the 03 parts of *P. santalinoides* has a moderate antiradical power compared to the control which was quercetin.

**Conclusion:**

In view of the different pharmacological activity recorded, the extract of the leaves should be recommended to treat patients suffering from gastroenteriditis.

## 1. Introduction

Medicinal plants have been used since ancient times as medicines for the treatment of human diseases [1]. About 80% of the inhabitants of developing countries, including Benin, still use plant resources such as medicinal plants to treat themselves [2]. These plant species are harvested in the wild or in domesticated form to treat bacterial diseases, especially toxi-infections and other associated disorders. *Pterocarpus santalinoides* is a species of the Fabaceae family known to have several uses in the daily life of local populations. Its leaves and fruits are used in human food as a vegetable and as a snack [3]. In traditional medicine, the leaves or the bark of the stem are used to treat gastroenteritis [4]. According to the works, extracts of *P. santalinoides* leaves are used in the treatment of diarrhea of bacterial and nonbacterial origin [5].

In recent years, there has been an increasing demand for the harvesting and use of the parts of this forest resource and the issue of availability and preservation of natural populations of this resource is of great concern. Recent studies conducted on the perception of local populations on the current availability of natural populations of *P. santalinoides* in southern Benin have highlighted the negative impact of anthropic activities on the latter [3]. This work also revealed that *P. santalinoides* is an endangered species in southern Benin with a vulnerability index Iv: 2.4 [6–9]. These studies linked anthropogenic factors such as the types of parts used (leaves, stem bark, root, fruits), the modes of collection, and the frequency of use of this species with the decline of its natural population already observed in the field. The work of Okoye et al. mentions that different parts of *P. santalinoides* are used in the treatment of parasitic diseases including malaria [7]. The aqueous macerate of the leaves is used in the treatment of chickenpox and measles. The boiled fruits are used to treat gastrointestinal ulcers [3]. The stem bark and roots are used orally and dermally to treat disorders related to inflammatory mechanisms, skin, respiratory, and sexual infections [3]. Odeh et al. showed that the leaves of *P. santalinoides* had a moderate antimicrobial activity against most of the pathogens involved in the development of several human pathologies [8]. Several other works, including those of Chic and Amom, have supported the ethnomedical applications of the leaves of this plant by attributing the pharmacological properties, particularly anti-inflammatory, of the species to its richness in flavonoids, alkaloids, terpenoids, saponins-glycosides, tannins, and vitamins. [3, 9]. While these different works have had the merit of documenting the factors that contribute to the regression of natural populations of *P. santalinoides*, they have not, however, proposed any practical measures that would give guidance on the most effective and appropriate organ to use to preserve the species from extinction. It is, thereforehvy,b imperative to undertake a comparative study on the pharmacological potential of the three main parts of *P. santalinoides* used in traditional medicine. This study aims to determine and compare the antimicrobial, anti-inflammatory, and antioxidant profile of the parts of *P. santalinoides*.

## 2. Materials and Methods

### 2.1. Plant Material and Extraction

The plant material consisted of roots, leaves, and stem bark of *P. santalinoides*. These parts of *P. santalinoides* were collected in April-May 2014 in the commune of Adjarra in southern Benin. The identification followed by the certification was carried out by Professor Hounnankpon YEDOMONHAN, the curator of the National Herbarium of the University of Abomey-Calavi, under the reference number DGB_21.

The samples were washed with clean water, dried in the shade, and ground into fine powder using an electric grinder (Brook Crompton series 2000, Germany). The time of harvest was chosen according to the foliage. Also, in this period, traditional healers were used to collect these plants for use according to Beninese Pharmacopoeia practices. Extraction was carried out by soaking the powder of each part in a water-ethanol solvent (80–20: v/v) under continuous stirring for 72 hours. The filtrate was dry and concentrated under vacuum in a rotary evaporator (Rota Vapor Re111, Germany) at 40°C. The yield (*R*) of the extracts was calculated according to the following formula:

(1) $$\begin{array}{c} R\left(\%\right)=\frac{\text{Me}}{\text{Mv}}\times 100, \end{array}$$

where *R* (%) is the yield in%; Me is the mass of the extract after solvent evaporation; and Mv is the mass of plant material used for extraction.

### 2.2. Experimental Animals

Male and female Wistar rats weighing (130–145 g), provided by Physiology/Pharmacology Department of the “University of Lomé” were used. They were housed in a standard environmental condition and fed with rodent standard diets and watered *ad libitum*. Animal care and handling conformed to international guidelines [10, 11]. All methods and protocols used in this study were observed following established public health guidelines “Guide for Care and Use of Laboratory Animals.”

### 2.3. Antibacterial Activity

Six bacterial strains including three wild strains (*E. coli* 0157H, *Salmonella* spp., and *S. aureus* sp.) and three reference strains (*E. coli* ATCC 25922, *S. aureus* ATCC 29213, *P. aeruginosa* ATCC 27853) were tested. The wild strains isolated from food were obtained from the Ministry of Health of Benin in the Section Hygiene Water and Sanitation.

Antibacterial potencies of the hydroethanolic extracts of *P. santalinoides* was assessed *in vitro* by determining the minimum inhibitory concentrations (MICs) and the minimum bactericidal concentrations (MBCs). Current tests were performed in 96-wells plates using the NCCLS procedure (2000) [12]. Twenty-four hour colonies were used to inoculate Mueller–Hinton broth. Suspensions were adjusted to 0.5 McFarland standard and subjected to dilution in Mueller–Hinton broth to get about 103 CFU/well. Negative control (well containing extracts and medium), and positive control (well containing medium and bacteria) were used in this test. Each clear wells were plated on nutrient agar to appreciate MBC. Thus, the lowest concentration that inhibit 99, 9% growth of bacteria corresponds to the MBC.

### 2.4. Preparation of Extract Solutions

From this powder, a hydroethanolic extraction was carried out according to the methodology described by Dosseh et al. [13]. Fifty grams of powder were macerated in 500 mL of the mixed solvent with equal volume of distilled water and 96% ethanol. The mixture was continuously stirred for 72 hours at room temperature (25°C). The homogenate obtained has been filtered three times on hydrophilic cotton and once on Wittman paper No. 3. The filtrate was dry and concentrated under vacuum in a rotary evaporator (Rota Vapor Re111, Germany) at 40°. The extract obtained has been weighed and used to evaluate the extraction yield (EY) and then kept in the refrigerator at 4°C. The total hydroethanolic extracts obtained were used to prepare solutions with a concentration of 50 mg/mL. These solutions were sterilized by filtering with a 0.22 *μ*m millipore membranes. The sterility of the extract solutions regarding bacterial and fungal strains was verified by inoculating aliquots of each solution on Mueller–Hinton agar then incubated at 37°C for 24 h.

The yield (*R*) of the extracts was calculated according to the following formula:

(2) $$\begin{array}{c} R\left(\%\right)=\frac{\text{Me}}{\text{Mv}}\times 100, \end{array}$$

where R (%) is the yield in %; Me is the mass of the extract after solvent evaporation; and Mv is the mass of plant material used for extraction.

### 2.5. Culture Media and Reagents

The medium Nutrient agar, Mueller–Hinton (agar and broth), and antibiotics discs were manufactured by Oxoid (Basingstoke, Hampshire, England). Folin-Ciocalteu reagent, quercetin, 2, 2-diphenyl-1picrylhydrazyl (DPPH), nitrite of sodium at 5%, and gallic acid were manufactured by Sigma Chemical (Saint-Louis, USA). All the other chemicals were analytical grade.

### 2.6. Antioxidant Activity of *P. santalinoides* Extracts

The antioxidant activity was assessed using the 2, 2-diphenyl-1picrylhydrazyl (DPPH) radical scavenging with quercetin as standard [14]. To perform each test, 250 *μ*L of the extracts or standard (50–100 mg/mL) dissolved in methanol were added to 1500 *μ*L of 100 *μ*mol/mL methanolic DPPH. [13] A blank was constituted with methanol. The mixtures were then incubated at room temperature for 30 min, and the absorbance was determined at 517 nm using spectrophotometer Thermo Scientic Genesys 10S UV-VIS. The assay was conducted in triplicate. The lowest absorbance represented the highest DPPH scavenging activity (SA). This effect was expressed as a percentage (%) calculated as follows:

(3) $$\begin{array}{c} \text{SA}\left(\%\right)=\frac{\text{Ac}-\text{Ae}}{\text{Ac}}\times 100, \end{array}$$

with SA (%) = percentage of inhibition; Ac = absorbance of the control solution; and Ae = absorbance in the presence of the extract or quercetin. IC_50_ values are calculated by the linear regression method from the curve (SA(%) = *f* (concentrations)). The tests were performed three times.

### 2.7. Quantification of Total Phenols

The total phenolic content (TPC) was determined according to the Folin-Ciocalteu procedure with gallic acid as standard. [12] A mixture of 10 mg of the lyophilized extracts dissolved in 10 mL of methanol to obtain the most concentrated solution (1 mg/mL) and 750 mL of Folin-Ciocalteu reagent were diluted 10 times in distilled water. The two solutions were allowed to stand for 5 min before to add 750 *μ*L of Na_2_CO_3_ (60%). After 90 min of incubation at room temperature in the dark, the absorbance against the prepared reagent blank was determined at 765 nm. The phenolic content was calculated as gallic acid equivalents (GAE)/g on the basis of standard curve of gallic acid.

### 2.8. Quantification of Total Flavonoids

Flavonoid quantification of the different hydroethanolic extracts of *P. santalinoides* part was done according to the method used by Dosseh et al. [13]. To 250 *μ*l of the extract (1 mg/mL) or standard (quercetin) prepared with methanol was added 0.4 mL of distilled water followed by 0.03 mL of 5% sodium nitrite solution dissolved in distilled water. After 5 min incubation at room temperature (25 ± 1°C), 0.02 mL of aluminum trichloride (10%) was added along with 0.2 mL of sodium carbonate solution (1 M). To the resulting reaction volume, 0.25 mL of distilled water was added. The absorbance was measured with a UV-visible spectrophotometer (Genesys 10S UV-Vis Spectrophotometer) at 510 nm against the “blank.” Total flavonoid content was deduced from a calibration range established with quercetin (0–500 *μ*g/mL), and results are expressed in *μ*g Quercetin Equivalent per mg dry extract (*μ*g QE/mg extract). The tests were performed in triplicate.

### 2.9. Evaluation of the Anti-Inflammatory Property

The anti-inflammatory activity was demonstrated by the carrageenan edema induction method described by Anani et al. [15]. For this test, the optimal doses of the plant extracts were determined.

### 2.10. Determination of the Optimal Doses of the Extract

Preliminary experiments were performed to determine the doses that would significantly prevent edema induced by injection under the plantar aponephrosis of carrageenan. Thirty minutes before the induction of plantar edema by injection of 0.1 mL of 1% carrageenan, four different doses (125, 250, 500, and 750 mg/kg) of each *P. santalinoides* extract were administered orally to different successive groups of five rats. The different doses were chosen based on the results of tests carried out on extracts of a species of the family that *Pterocarpus erinaceus*.

### 2.11. Induction and Determination of Edema Volume

Adult Wistar rats of both sexes weighing between 130 and 145 g were randomized into four groups of five rats. These rats were fasted for 12 h before experimentation. Two groups of controls were constituted. These were group 1: negative control which received carrageenan without being treated and group 2: positive control which was treated with the reference product. Thirty minutes before the injection of carrageenan, the test rats received orally 250 and 500 mg/kg of hydroethanolic extracts of each part of *P. santalinoides*. Acetylsalicylic acid (ASA) was used as the reference product at the dose of 300 mg/kg. The negative control group received 0.9% NaCl isotonic saline (10 mL/kg).

Paw volume was determined by immersion in a tube containing distilled water. The introduction of the paw causes an increase in the water level which is brought back to its initial level using two insulin syringes combined with the tube containing distilled water [13]. The volume of the paw corresponding to the amount of water displaced was read directly from the 1 mL syringes. The volume of edema VT at a given time *t* is as follows:

(4) $$\begin{array}{c} \text{VT}=V_{t}-V_{0}, \end{array}$$

with *V*_0_ is thethe initial paw volume and *V*_*t*_ is the the paw volume at time *t* after carrageenan injection and extract treatment.

The percentage of inhibition (% Inh) of edema is calculated according to the formula of Jain et al. [16]:

(5) $$\begin{array}{c} \%\text{Inh}=\frac{V_{t}\text{control}-V_{t}\text{extract}}{V_{t}\text{control}}\times 100, \end{array}$$

with *V*_*t*_control is the volume of edema in negative control rats and *V*_*t*_extract is the volume of edema in rats treated with *P. santalinoides* extract or ASA.

### 2.12. Statistical Analyses

Means and standard deviations were calculated using the Excel spreadsheet. Simple univariate analysis of variance (ANOVA) followed by Tukey's multiple comparison was performed using GraphPad Prism software to reveal significant differences in mean percentages of increase and/or inhibition.

## 3. Results

The extraction yield of the three extracts was 6%, 1%, and 2% for the leaf, stem bark, and root extract, respectively.

### 3.1. Antibacterial Activity of Hydroethanolic Extracts of *P. santalinoides* Parts

The MICs and MBCs of the tested bacteria are recorded in Table 1. The parts of *P. satalinoides* showed various degrees of antibacterial activity against the tested strains.

The MICs of the leaf extracts varied from 6.25 to 25 mg/mL for all strains. For the stem bark extracts, MICs ranged from 6.5 to 25 *μ*g/mL for five strains (*E. coli* 0157H, *S. aureus* ATCC 25922, *Salmonella* sp., *E. coli* ATCC 25922, and *P. aeruginosa* ATCC 27853) and 3.125 mg/mL for *S. aureus*. As for the root bark extracts, the MICs varied from 12.5 to 50 mg/mL.

The leaf MBC values were 6.25 for the wild-type *Staphylococcus* strain (*S. aureus* ATCC 25922) and 25 *μ*g/mL to 100 mg/mL for the other 4 strains (*E. coli* 0157H, *Salmonella* sp., *E. coli* ATCC 25922, and *P. aeruginosa* ATCC 27853). The stem bark extracts showed variable MBCs against the studied strains with a value of 6.25 mg/mL for the reference strain of *Staphylococcus* (*S. aureus* ATCC 25922). The root extracts exercised bactericidal activity on only two strains with MBCs ranging from 25 to 100 mg/mL (Table 1). Leaf extracts showed bactericidal effect on four strains (66.66%) and bacteriostatic effect on two strains (33.33%). As for the root extracts, a bactericidal effect on one strain (1.66%) and a bacteriostatic effect on five strains (83.33%).

As for the extracts of stem bark, it showed a bactericidal effect on the six strains under this study, that is, 100% (Table 1).

### 3.2. Antioxidant Power of Hydroethanolic Extracts of *P. santalinoides*

The antiradical power of each extract is as follows: 2.18% stem bark extract (EHETr) (IC50 = 45.83 ± 1.67 *μ*g/mL) is the highest, followed by 0.9% for leaf extract (EHEFe) (IC50 = 110 ± 0.29 *μ*g/mL). The potency of the root extract is 0.54% (IC50 = 184.16 ± 0.33 *μ*g/mL). These antiradical powers increase in correlation with the concentration of the extract of each part. The DPPH test showed that the hydroethanol extract of the parts (leaves, stem bark, and root) of *P. santalinoides* has moderate antiradical power compared to the control, which is quercetin (Table 2).

### 3.3. Total Phenol Content

Total phenol compounds, as determined by Folin-Ciocalteu method, are reported as gallic acid equivalents by reference to standard curve (*y* = 0,005018*x* + 0,05519; *r*_2_ = 0,9925). The total phenol content of hydroethanolic total extracts varies from 146.23 to 309.14 *μ*g·EAG/mg of extract (Table 2). Indeed, the stem bark (309.14 ± 1.54 *μ*g·EAG/mg of extract) concentrates more polyphenols followed by the leaves (209.34 ± 4.88 *μ*g·EAG/mg extract) and the root (146.23 ± 4.21 *μ*g·EAG/mg extract).

### 3.4. Total Flavonoid Content

The total flavonoid content varies from one plant extract to another (Table 2). The stem bark records the highest content (301.72 ± 0.47 *μ*g·EQ/mg extract) followed by the leaves (190.27 ± 1.35 *μ*g·EQ/mg extract) by reference to standard curve (*y* = 6.328*x* + 0.693, *r*_2_ = 0.9391). Root bark has the lowest concentration (152.02 ± 1.58 *μ*g·EQ/mg extract) of total flavonoids.

### 3.5. Anti-Inflammatory Activity of Hydroethanol Extracts of *P. santalinoides*

The maximum inflammatory effect on the paw occurred 5 h after carrageenan administration for a value of 78.00 ± 4 mL (Table 3).

The anti-inflammatory activity of the reference product (acetylsalicylic acid) was 55.4 ± 4 mL at the end of the experiment, corresponding to 25.97% inhibition after carrageenan administration.

Extracts from the different *P. santalinoides* showed varying degrees of edema inhibition. The results of the test indicate that the hydroethanolic extracts of the leaves showed significant dose-dependent anti-edema activity from the 4th and 5th hour, that is, inhibition percentages of 26.72% and 28.20%, respectively (Table 3). As for the stem bark extract at the same dose (500 mg/kg), a dose-dependent inhibition was recorded from the 1^st^, 2^nd^, and 3^rd^ hour to start decreasing from the 4th until the end of the experiment. This anti-edema activity is reflected in the respective inhibition percentages of 36.09%, 38.98%, and 39.50% (Table 3). The hydroethanolic extract of the root administered at a dose of 250 mg/kg caused an increasing and significant (*p* < 0.05) inhibition compared to the control from the 3rd to the 5th hour. Its best anti-edema activity was obtained at the 500 mg/kg dose from the 2nd hour with a percentage of inhibition of 31.86% (Table 3).

## 4. Discussion

The analysis of the results concerning the antimicrobial activity of the parts of *P. santalinoides* indicates that all the tested bacterial strains are sensitive to the different extracts. However, these extracts inhibited the growth of the tested microorganisms to different degrees in vitro. These data indicate that the leaf and stem bark extracts are more active on the strains under study with MICs ranging from 6.25 to 25 mg/mL for the leaves and from 3.125 mg/mL to 25 mg/mL for the stem bark. Their antibiotic powers expressed in percentage are 66.66% and 100% against a bacteriostatic effect of 33.33%. These results are in agreement with the work of Chic and Amom [17] who associate the antibiotic powers of the leaves with the major chemical groups they contain. As for the trunk bark extract, it is the first time it has been studied. The root extract is the least active with a bactericidal power of 16.67% on *S. aureus*. These differences in activity can be explained by the fact that these extracts do not contain the same phytochemical components. These extracts contain different concentrations of phytochemicals [8, 18, 19]. The work of Falleh et al. has already reported this unequal distribution of secondary metabolites in different parts of the same plant [20]. According to Ndjonka et al., the antimicrobial potential of an extract depends on its composition in tannins, flavonoids, saponosides, and alkaloids [21]. These results obtained are comparable to those presented by Anowi et al. who showed that the phytochemical composition of the methanolic extract of *P. santalinoides* leaves was responsible for the observed analgesic activity [22].

Antioxidant assays showed that the inhibitory concentrations (IC50) of the extracts of the different parts ranged from 45.83 to 184.16 *μ*g/mL of extract. The stem bark extract showed the highest IC50 (45.83 ± 1.67 *μ*g/mL), followed by the leaf extract (110 ± 0.29 *μ*g/mL). The IC50 of the root is equal to the IC50 = 184.16 ± 0.33 *μ*g/mL. In addition, its total phenol and flavonoid content is higher than leaf and root extracts of leaf and root extracts, respectively. The DPPH test showed that the hydroethanol extract of the parts (leaves, stem bark and root) of *P. santalinoides* has a moderate antiradical power compared to the control which is quercetin. This power increases in correlation with the concentration of the extract of each parts.

This antiradical property of the tested parts of *P. santalinoides* could be related to the presence of bioactive compounds such as total phenols and total flavonoids observed. According to the work of Dosseh et al., phenolic compounds are responsible for antioxidant activities. [13] The proportions of flavonoids in the leaf extracts are largely higher than those obtained by Anowi et al. who had shown that the flavonoid content of the aqueous extract of *P. santalinoides* leaves was about 6.7 ± 0.02 *μ*g·EAG/mg of extract, that is, 3.53 times lower [22, 23]. This large difference could be explained, on the one hand, by the nature of the extraction solvent and, on the other hand, by the geographical origins and climatic conditions that prevailed during the collection of the plant material used for the experiments. As for the extracts of the bark of the stem and the roots, these values remain the first to be determined. It appears from the anti-inflammatory test that the inflammation obtained from the 1st h until the 5th h carrageenan produced a phlobogenic effect, inducing a maximum volume of edema after 5 h. [12] According to Anani et al. [15], carrageenan causes local inflammation when injected into the fascia of the foot sole [24]. The cause of this inflammatory reaction is tissue damage. This tissue injury induces the synthesis of histamine, prostaglandins, leukotrienes Ammon et al. [25], platelet activating factor (PAF), cytokines, nitric oxide (NO), and tumor necrosis factor (TNF) [13, 26].

Hydroethanol extracts of the leaves, stem bark, and roots of *P. santalinoides* administered at a dose of 500 mg/kg body weight variably inhibited plantar edema in rats. This inhibitory effect started from the 4th hour and persisted until the 5th hour for the leaf extract. This delayed effect of the extracts' activity was previously reported by Amezouar et al. with the ethanolic extract of Erica arborea at doses of 200 and 400 mg/kg body weight which significantly (*p* < 0.05) prevented plantar edema in rats only from the 2nd hour of treatment [27].

As for stem bark extract, the anti-inflammatory effect induced by this material reached its optimal level at the 1^st^, 2^nd^, and 3^rd^ hour before regressing from the 4th hour. This inhibition of the anti-inflammatory effect is justified by its concentration in bioactive compounds (tannins, polyphenols and flavonoids). These results are in agreement with those obtained by Akkol et al. who had linked these observations to the identified phytochemicals [28]. This extract exerts a short-term anti-inflammatory effect. The administration of root bark extract which induced an inflammation inhibitory effect at the dose of 250 mg/kg from the 3rd hour to the 5th hour indicates that this effect increases with time. These results indicate that the effect of hydroethanol extract of *P. santalinoides* root bark is dose-dependent.

## 5. Conclusion

This comparative study of some biological activities of hydroalcoholic extracts of *P. santalinoides* confirmed the antimicrobial properties of the parts (leaf, stem bark and root) of *P. santalinoides* on the bacteria responsible for gastroenteritis. The results also showed that the hydroalcoholic extracts of leaves and root showed the best antibacterial, anti-inflammatory, and antioxidant activities. In addition to activity between the extracts tested, and in relation to the conservation of the species, the extracts of the leaves must be privileged for the daily uses of the populations using this resource.

## Figures and Tables

**Table 1:** Action of hydroethanolic extracts of *P. santalinoides* organs on the *in vitro* growth of six bacterial species.

Hydroethanolic extracts	Strain	MIC (mg/ml)	MBC (mg/ml)	Antibacterial effects
Leaves	*E*. *coli* 0157H	25	100	Bactericidal
	*S. aureus*	6.25	6.25	
	*S. aureus* ATCC 25922	6.25	12.5	
	*Salmonella* sp	25	25	
	*E*. *coli* ATCC 25922	6.25	100	Bacteriostatic
	*P. aeruginosa* ATCC 27853	12.5	100	

Stem bark	*E. coli* ATCC 25922	25	100	Bactericidal
	*Salmonella* sp.	12.5	25	
	*P. aeruginosa* ATCC 27853	12.5	25	
	*S. aureus* ATCC 25922	3.125	6.25	
	*S. aureus*	25	25	
	*E. coli* 0157H	25	25	

Root bark	*E. coli* 0157H	50	>100	Bacteriostatic
	*E. coli* ATCC 25922	25	>100	
	*Salmonella* sp	50	>100	
	*P. aeruginosa* ATCC 27853	12.5	>100	
	*S. aureus* ATCC 25922	12.5	100	
	*S. aureus*	25	25	Bactericidal

MIC: minimum inhibitory concentration; MBC minimum bactericidal concentration.

**Table 2:** Evaluation of free radical scavenging activity (IC50) and quantification of total phenols and total flavonoids in hydroethanolic extracts of *P. santalinoides*.

Extracts	IC_50_ (a)	Total phenols (b)	Total flavonoids (c)
Leaves	110 ± 0.29	209.34 ± 4.88	190.27 ± 1.35
Stem bark	45.83 ± 1.67	309.14 ± 1.54	301.72 ± 0.47
Root bark	184.16 ± 0.33	146.23 ± 4.21	152.02 ± 1.58
Quercetin	8.57 ± 0.15	—	—

(a): *μ*g/ml; (b): *μ*g of gallic acid equivalent per mg extract (*μ*g.EAG/mg of extract); (c): *μ*g of quercetin equivalent per mg extract (*μ*g.EQ/mg extract).

**Table 3:** Effect of different doses of hydroethanolic extracts of *P. santalinoides* organs on edema volume.

Treatment (mg/kg)		Edema volume (mL) (%)				
		1 h	2 h	3 h	4 h	5 h
Saline		0.43 ± 0.02	0.59 ± 0.02	0.64 ± 0.03	0.70 ± 0.02	0.78 ± 0.04
EHEFe	250	0.39 ± 0.02 (10.79%)	0.48 ± 0.08 (16.94%)	0.59 ± 0.04 (7.81%)	0.66 ± 0.04 (5.17%)	0.70 ± 0.04 (5.12%)
	500	0.35 ± 0.03 (17.84%)	0.480 ± 0.02 (18.64%)	0.492 ± 0.03 (23.43%)	0.513 ± 0.01^*∗*^ (26.72%)	0.48 ± 0.01^*∗*^ (28.20%)

EHETr	250	0.38 ± 0.08 (10.79%)	0.53 ± 0.07 (10.17%)	0.57 ± 0.07 (10.93%)	0.56 ± 0.07 (14.59%)	0.60 ± 0.09 (16.02%)
	500	0.27 ± 0.04^*∗*^ (36.09%)	0.36 ± 0.03^*∗∗*^ (38.98%)	0.40 ± 0.03^*∗∗*^ (39.50%)	0.40 ± 0.02^*∗*^ (29.52%)	0,51 ± 0.04^*∗*^ (28.84)

EHERa	250	0.381 ± 0.04 (10.79%)	0.490 ± 0.05 (25.42%)	0.491 ± 0.05^*∗*^ (28.12%)	0.442 ± 0.06^*∗*^ (29.59%)	0.423 ± 0.09^*∗*^ (35.89%)
	500	0.341 ± 0.08 (20.18%)	0.420 ± 0.03^*∗*^ (28.81%)	0.372 ± 0.04^*∗∗*^ (42.18%)	0,291 ± 0.07^*∗∗∗*^ (58.33%)	0.170 ± 0.07^*∗∗∗*^ (78.20%)

ASA	300	0.31 ± 0.03^*∗*^ (26.76%)	0.40 ± 0.03^*∗∗*^ (31.86%)	0.44 ± 0.04^*∗∗*^ (30.94%)	0.54 ± 0.04^*∗*^ (22.13%)	0.55 ± 0.04^*∗*^ (25.97%)

Values are mean ± SD and the percentage inhibition of paw edema carrageenan-induced is given within parenthesis; EHEFe: hydroethanolic extracts of *P. santalinoides* leaves; EHETr: hydroethanolic extracts of *P. santalinoides* stem bark; EHERa: hydroethanolic extract of the roots; ASA: acetylsalicylic acid; ^*∗*^*p* < 0.05 and ^*∗∗*^*p* < 0.01: significant difference from control group; SD: standard deviation.

## Data Availability

No underlying data were used to support the results of your study.
